# Hygroscopic properties of potassium chloride and its internal mixtures with organic compounds relevant to biomass burning aerosol particles

**DOI:** 10.1038/srep43572

**Published:** 2017-02-27

**Authors:** Bo Jing, Chao Peng, Yidan Wang, Qifan Liu, Shengrui Tong, Yunhong Zhang, Maofa Ge

**Affiliations:** 1 State Key Laboratory for Structural Chemistry of Unstable and Stable Species, Beijing National Laboratory for Molecular Sciences (BNLMS), Institute of Chemistry, Chinese Academy of Sciences, Beijing 100190, P. R. China; 2 University of Chinese Academy of Sciences, Beijing 100049, P. R. China; 3 The Institute of Chemical Physics, School of Chemistry, Beijing Institute of Technology, Beijing 100081, P. R. China; 4 Center for Excellence in Regional Atmospheric Environment, Institute of Urban Environment, Chinese Academy of Sciences, Xiamen 361021, P. R. China

## Abstract

While water uptake of aerosols exerts considerable impacts on climate, the effects of aerosol composition and potential interactions between species on hygroscopicity of atmospheric particles have not been fully characterized. The water uptake behaviors of potassium chloride and its internal mixtures with water soluble organic compounds (WSOCs) related to biomass burning aerosols including oxalic acid, levoglucosan and humic acid at different mass ratios were investigated using a hygroscopicity tandem differential mobility analyzer (HTDMA). Deliquescence points of KCl/organic mixtures were observed to occur at lower RH values and over a broader RH range eventually disappearing at high organic mass fractions. This leads to substantial under-prediction of water uptake at intermediate RH. Large discrepancies for water content between model predictions and measurements were observed for KCl aerosols with 75 wt% oxalic acid content, which is likely due to the formation of less hygroscopic potassium oxalate from interactions between KCl and oxalic acid without taken into account in the model methods. Our results also indicate strong influence of levoglucosan on hygroscopic behaviors of multicomponent mixed particles. These findings are important in further understanding the role of interactions between WSOCs and inorganic salt on hygroscopic behaviors and environmental effects of atmospheric particles.

Atmospheric aerosols have important impacts on the earth’s climate through direct and indirect radiative forcing^1^. The magnitude of climate effects significantly depends on the hygroscopic properties of aerosol particles. Water uptake can control the particle size, phase state, chemical and optical properties of aerosol particles. The atmospheric aerosols are generally complex mixtures consisting of inorganic and organic compounds. The total organic mass contributes to 10–90% of atmospheric fine particles^2^,^3^, and a large fraction of organics is water soluble organic compounds (WSOCs)^4^. It has been found that WSOCs strongly affect the hygroscopic behavior of inorganic components and may promote water uptake of aerosol particles under low relative humidity (RH) conditions^5^. Further study on the hygroscopicity of WSOCs and inorganic salt mixtures could contribute to enhanced understanding of the environmental effects of atmospheric aerosols.

Biomass burning is an important emission source of global atmospheric trace constituents and aerosol particles, resulting in air pollution affecting regional and local air quality, and atmospheric chemistry^6^,^7^,^8^. Additionally, biomass burning aerosols can scatter and absorb solar radiation as well as act as cloud condensation nuclei (CCN), which could affect the global radiation budget^8^,^9^. Primary aerosol particles originating from biomass burning are mainly composed of black carbon, inorganic species, and organic material. The black carbon is assumed to be nonhygroscopic and thus the water uptake of biomass burning aerosols is primarily contributed by inorganic and organic components^10^,^11^. A large fraction of inorganic components from biomass burning is composed of potassium salts such as potassium chloride (KCl), potassium sulphate (K_2_SO_4_) and potassium nitrate (KNO_3_). Generally, potassium and chloride are the most abundant ions from the biomass smoke emissions^12^,^13^. Potassium salts generated from fresh biomass burning aerosols typically exist as KCl^12^, which can undergo heterogeneous reaction with atmospheric acid gas leading to the formation of potassium sulfate and potassium nitrate during the aging process^14^,^15^. Compared to potassium sulfate and potassium nitrate, KCl has a lower deliquescence relative humidity (DRH) and stronger hygroscopicity after deliquescence^16^,^17^. The organic matter in the biomass burning particles contains a considerable amount of WSOCs, which are usually divided into three classes including mono/dicarboxylic acids, neutral compounds and polycarboxylic acids^18^. Oxalic acid has been identified as the dominant dicarboxylic acid in aerosol particles^19^. Levoglucosan as the proxy of neutral compounds has been recognized as the major constituents of pyrolysis products, as well as a tracer for biomass burning^20^. The polycarboxylic acids also known as humic-like substances (HULIS), contribute up to 40% for the WSOCs^21^. Due to similar structural features, the humic or fulvic acids are generally used as the model species for HULIS.

Numerous laboratory studies have focused on the hygroscopicity of WSOCs and related internal mixtures with inorganic salt mainly involving ammonium sulfate or sodium chloride^22^,^23^,^24^,^25^,^26^,^27^,^28^,^29^. It was found that the effects of WSOCs on the deliquescence behavior of inorganic salt depended on the properties of organics^30^. Chan and Chan found that the same WSOC may have various impacts on the water uptake of mixed particles after full deliquescence of ammonium sulfate or sodium chloride^31^. Previous studies revealed that strong interactions between oxalic acid and inorganic salt in the atmosphere may result in the formation of organic salts, thus affecting the composition and hygroscopic behaviors of atmospheric particles^32^,^33^. Considering the unique chemical constituents of biomass burning aerosols as well as potential interactions between species, it is vital to investigate how such specific compositions affect the hygroscopic properties of aerosol particles.

Here the hygroscopic behaviors of potassium chloride and its mixtures with water soluble organic compounds including oxalic acid, levoglucosan and humic acid relevant to biomass burning are determined using the hygroscopicity tandem differential mobility analyzer (HTDMA). The effects of WSOC composition content on hygroscopic properties of aerosol particles are studied. The measurement results are compared with predictions from Zdanovskii-Stokes-Robinson (ZSR) method and Aerosol Inorganic-Organic Mixtures Functional groups Activity Coefficients (AIOMFAC) model. The influence of interactions between inorganic salt and organic compounds on the hygroscopic growth is also evaluated.

## Results and Discussion

### Pure Substances

**Figure 1:**
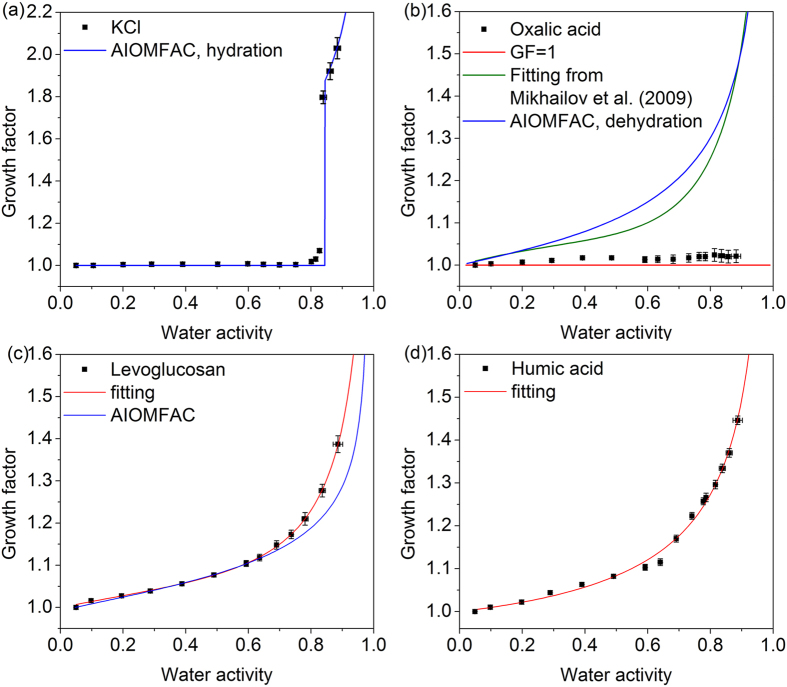
Hygroscopic growth factors of 100 nm potassium chloride (KCl) (**a**), oxalic acid (**b**), levoglucosan (**c**) and humic acid (**d**) particles as a function of water activity. The prediction curve from the AIOMFAC is presented for KCl, oxalic acid and levoglucosan particles. The fitting curve for oxalic acid is from Mikhailov *et al*.^37^. For levoglucosan and humic acid particles, the fitting curve is based on our measurement. The uncertainty in our measured RH and growth factors represented by standard deviation is typically within ±1.5% and 0.02, respectively.

The hygroscopic growth of 100 nm KCl particles was determined first. As shown in Fig. 1a, the deliquescence transition of KCl particles occurred around 84% RH, which was in good agreement with reported values of 85% RH and 83% RH in the literature^16^,^34^. Additionally, the hygroscopic growth factors (GFs) of 100 nm KCl particles were 1.80 and 2.05 at 85% and 90% RH, respectively, which was similar to the literature values of 1.9 and 2.1 at corresponding RH^34^. The predicted deliquescence point from AIOMFAC model at 298 K is 84.5% RH, highly consistent with our experimental value. It is obvious that the hygroscopic growth of KCl particles over the whole RH range is well described by the AIOMFAC model. Carrico *et al*.^34^ also found the measured hygroscopic growth of 100 nm KCl particles could compare favorably to the theory curve based on water activity data from Tang^35^ with the assumption of spherical dry and wet particles. Due to the treatment of KCl in AIOMFAC based on fitting data from Tang^35^ and others, it can be expected that predicted hygroscopic growth factors for KCl from AIOMFAC agree well with those from Tang^35^,^36^.

The hygroscopic growth of WSOCs can be seen in Fig. 1b,c and d. The predicted curve from AIOMFAC is a dehydration curve assuming oxalic acid remains liquid in the whole RH range, which is used to compare with the fitting curve from Mikhailov *et al*.^37^. The oxalic acid particles showed no obvious water uptake or deliquescence phase transition over the RH range studied, consistent with previous observations using electrodynamic balance (EDB) and other measurement methods^23^,^32^. This phenomenon can be expected from the deliquescence point for oxalic acid crystalline up to 97% RH, as suggested by theory prediction and bulk measurement^23^. However, some HTDMA studies found 100 nm oxalic acid particles could take up water far below the deliquescence point due to the initial state of oxalic acid in their measurements in an amorphous or highly concentrated liquid phase^37^,^38^, as also indicated by the fitting curve from Mikhailov *et al*.^37^. During the HTDMA measurements, oxalic acid may exist as dihydrate in the initial particles, which could account for no evaporation losses^37^,^38^,^39^. The observation of negligible hygroscopic growth below deliquescence point suggests oxalic acid particles generated in the present study tend to be in a crystalline state. The discrepancy between phase state of oxalic acid in our study and Mikhailov *et al*. is likely due to dry conditions and electric charge effects. Mikhailov *et al*. have pointed out that the microstructure and morphology of aerosol particles during HTDMA measurements can be affected by the rate of drying as well as electric charge effects^37^. In their study, the aerosol droplets generated by nebulisation were dried using a couple of silica gel diffusion dryers (SDD) and then the dry aerosols were charged by a neutralizer (^85^Kr, TSI, 2 mCi, Model 3077). In our study, aerosol particles flowed through a silica gel diffusion dryer (SDD) and a Nafion gas dryer (Model PD-100T-24MSS, Perma Pure Inc., USA) to be dried and then were charged in an electrical ionizer by corona discharge in high voltage electric field.

As for levoglucosan, the 100 nm particles presented continuous hygroscopic growth without deliquescence transition from low RH, which was in agreement with early observations^27^,^40^,^41^. The submicron levoglucosan particles may exist in a form of highly concentrated droplet at low RH, as indicated by the continuous hygroscopic growth over the RH range studied. The measured GFs of levoglucosan at 80, 85, and 90% RH were 1.21, 1.28, and 1.39, respectively. This was consistent with reported values (GF = 1.18, 1.23, and 1.38 at 80, 85, and 90% RH, respectively)^40^, which were also close to the measurements by Koehler *et al*.^41^ and Svenningsson *et al*.^27^. It can be seen that the predicted growth factors from AIOMFAC model for oxalic acid were in fair agreement with the fitting data in the literature at high RH but far too high at moderate RH while for levoglucosan the predictions underestimated the measurements at high RH. The considerable discrepancies between experimental results and model estimations could be attributed to inappropriate group interaction parameters applied in the model. The molecular structure of organics like levoglucosan with several polar functional groups in close vicinity may lead to relatively strong intramolecular interactions, which is not taken into account by the AIOMFAC^42^. For humic acid (Aldrich), the continuous water uptake was also observed from low RH with increasing RH. The measured GFs at 85 and 90% RH were 1.33 and 1.45, respectively. Brooks *et al*.^25^ observed the GFs of Fluka humic acid were 1.49 and 1.66 at 85 and 90% RH, respectively. Various HTDMA studies have measured GFs between 1.05 and 2.00 in the 85–95% RH range for humic and fulvic acids^43^.

### Potassium Chloride/Oxalic Acid Mixtures

**Figure 2:**
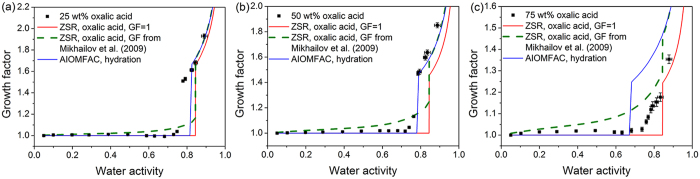
Hygroscopic growth factors of mixed aerosols containing potassium chloride and oxalic acid with various mass ratio (**a**) 3:1, (**b**) 1:1 and (**c**) 1:3 as a function of water activity. Hygroscopic growth curves from the ZSR and AIOMFAC model are indicated by red and blue solid lines, respectively. The green dashed line represents the ZSR predictions based on GFs of oxalic acid from Mikhailov *et al*.^37^.

The hygroscopic behaviors of mixed particles containing KCl and oxalic acid with different mass ratios are shown in Fig. 2. When the mass fraction of oxalic acid was 25%, this mixture did not show hygroscopic growth below 75% RH and the GF suddenly increased to greater than 1.5 at 79 ± 1% RH, indicating the deliquescence transition occurred. Although oxalic acid accounts for a small fraction in this mixture, it still could strongly affect the deliquescence behavior of KCl, as indicated by the reduction of DRH from 84% to 79%. The hygroscopic growth of mixed particles at high RH above 80% was well reproduced by the AIOMFAC predicted curve. The predicted DRH from AIOMFAC model was 82.6% RH slightly greater than the measurement result, seen in Table 1. Asfor the ZSR method, the predictions based on GFs of oxalic acid from Mikhailov *et al*.^37^ were more close to the measured values at high RH. In the case of equal mass mixed particles (Fig. 2b), the complete deliquescence was observed at 79 ± 1% RH, which was in good agreement with the AIOMFAC predicted value of 79% RH. The ZSR relation based on GF = 1 for oxalic acid greatly underestimated the water content of 1:1 KCl/oxalic acid particles at high RH. This discrepancy should be caused by the fact that solid oxalic acid is dissolved in the water absorbed by KCl after its full deliquescence thus contributing to water uptake of mixed particles. It was also confirmed by the ZSR predictions based on GFs for oxalic acid from Mikhailov *et al*.^37^, which were more close to the measurement results. Previous studies also showed that the ZSR method could underestimate the hygroscopic growth of mixed particles without taking partial or complete dissolution of components with limited solubility into consideration^27^,^44^. The measured DRH seems to be insensitive to mass fraction of oxalic acid. The similar phenomenon was also reported in the literature. The early study by Cruz and Pandis showed the measured DRHs of ammonium sulfate or sodium chloride internally mixed with glutaric acid remained unchanged within the uncertainty of the measurements for 20%, 50% and 80% mass fraction of the acid^22^. In addition, the good agreement in measured DRH of ammonium sulfate/oxalic acid mixture for 50% and 4.2% mass fraction of oxalic acid also indicates the negligible effects of oxalic acid content on the deliquescence point of inorganic salts^39^,^45^.

Figure 2c shows the hygroscopic growth of 100 nm 1:3 KCl/oxalic acid mixed particles. A slight hygroscopic growth below 70% RH was observed and after that the particles continued to take up water without experiencing deliquescence transition prior to 90% RH. A sudden increase of GF was observed at 88–90% RH, indicating the emergence of phase transformation. As for this mixing ratio, both ZSR and AIOMFAC methods failed to capture the hygroscopic behaviors including the deliquescence point and growth factors. This suggests that interactions between KCl and oxalic acid may result in the discrepancies for water content between measurement results and model predictions. Previous studies have shown the formation of organic salts may considerably alter the hygroscopicity of mixed particles^33^,^44^. Ma *et al*.^32^ observed that water uptake by internal oxalic acid/sodium chloride (molar ratio 1:2) mixture was substantially lower than that predicted by ZSR rule at high RH, caused by formation of less hygroscopic sodium oxalate resulting from evaporation of HCl during drying process. It has been found that sodium oxalate exhibited no hygroscopic growth even at 90% RH observed by EDB or HTDMA technology^44^,^46^. For KCl/oxalic acid mixed systems, it seems that the influence of chloride depletion on the hygroscopic growth is significant only when oxalic acid content is dominant in the mixed particles. Ghorai *et al*.^47^ investigated hygroscopic properties of mixed particles containing NaCl/dicarboxylic acid (malonic acid or glutaric acid) at different mixing ratio. They found that chloride depletion was enhanced with increasing organic acid content in the particles using X-ray elemental analysis. Similarly, a significant fraction of KCl in the 1:3 KCl/oxalic acid mixture was transformed into less hygroscopic potassium oxalate due to evaporation of HCl during drying process, thus resulting in reduction of water uptake by mixed particles. The hygroscopic behaviors of 100 nm potassium oxalate particles were determined using our HTDMA. The measured deliquescence point was around 89% RH and the measured GF was 1.71 at 90% RH, which was different from the property of sodium oxalate. This may explain the phenomenon of the substantial increase of GF at 90% RH for 1:3 KCl/oxalic acid mixed particles. Considering the model methods do not take the chemical reaction (R1) into consideration, the resulting composition change of the particles has consequences for the water uptake predictions.


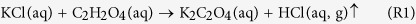


The composition changes in the particles were not taken into account in the AIOMFAC, leading to obvious deviation between predicted and measured GFs of the deliquesced particles. As for KCl aerosols with 25 or 50 wt% oxalic acid, the predictions were comparable to the measurements for deliquesced particles due to no significant composition changes.

### Potassium Chloride/Levoglucosan Mixtures

**Figure 3:**
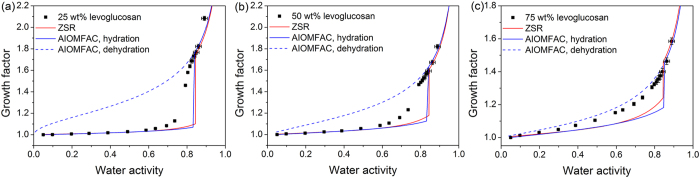
Hygroscopic growth factors of mixed aerosols containing potassium chloride and levoglucosan with various mass ratio (**a**) 3:1, (**b**) 1:1 and (**c**) 1:3 as a function of water activity. Hygroscopic growth curves from the ZSR and AIOMFAC model are indicated by red and blue lines, respectively. The predicted growth curve from AIOMFAC upon dehydration is indicated by blue dash line.

Figure 3 shows the hygroscopic growth of 100 nm KCl/levoglucosan mixed aerosol particles with increasing RH. The 3:1 KCl/levoglucosan mixed particles deliquesced at 80 ± 1% RH, slightly lower than the predicted value 84% RH by AIOMFAC. The predicted hydration curves from ZSR and AIOMFAC model were consistent with measured growth tendency above 84% RH. For 1:1 KCl/levoglucosan mixed particles, the measured deliquescence point occurred at 79 ± 1% RH compared to AIOMFAC predicted 84.3% RH. The hygroscopic growth of mixed particles was well described by both model methods above predicted deliquescence point. Parsons *et al*.^48^ investigated deliquescence behavior of ammonium sulfate/levoglucosan mixed particles with a variety of composition ratio. They reported the deliquescence point of ammonium sulfate decreased with increasing organic content. When the mass fraction of levoglucosan in KCl aerosols increased to 75%, the particles presented gradual water uptake without obvious phase transition over the whole RH range studied. It was obvious that hydration predictions from ZSR and AIOMFAC markedly underestimated water uptake at medium RH. Previous studies have revealed that submicron levoglucosan particles may exist in a form of highly concentrated droplet at low RH, indicating the strong water retention ability of this species^37^,^40^. In the case of 1:3 KCl/levoglucosan mixed particles, minor KCl component could be partially dissolved in the water absorbed by major levoglucosan component thus contributing to water uptake at medium RH. To better reproduce the measured hygroscopic growth, the dehydration predictions from AIOMFAC model assuming individual component in a liquid state in the whole RH range were also shown in Fig. 3. It can be seen that predictions were slightly larger than the measurement results for 1:3 KCl/levoglucosan mixture, which may suggest KCl was not completely dissolved in aqueous phase at medium RH. Ling and Chan^49^ also observed partial deliquescence phenomenon at intermediate RH for malonic acid/ammonium sulfate mixed particles.

### Potassium Chloride/Humic Acid Mixtures

**Figure 4:**
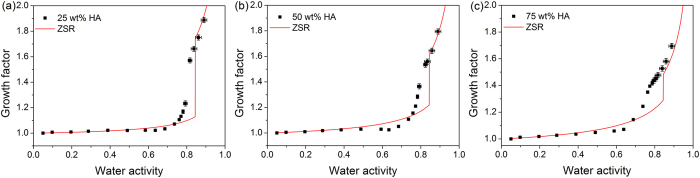
Hygroscopic growth factors of mixed aerosols containing potassium chloride and humic acid (HA) with various mass ratio (**a**) 3:1, (**b**) 1:1 and (**c**) 1:3 as a function of water activity. Hygroscopic growth curves from the ZSR method are indicated by red solid lines.

Humic acid is not incorporated into AIOMFAC model due to its complex composition. Thus, only ZSR predicted curves are shown to compare with measured growth factors for mixtures containing humic acid. The hygroscopic growth of 100 nm particles containing KCl and humic acid with different mass ratio is shown in Fig. 4. For 3:1 KCl/humic acid mixture, an obvious deliquescence point was observed at 80% RH, lower than that of pure KCl. The ZSR relationship can accurately predict the hygroscopic growth at low and high RH. The 1:1 KCl/humic acid mixed particles showed a deliquescence point around 80% RH and the water uptake was well predicted by the ZSR rule after full deliquescence of KCl. In the case of 1:3 KCl/humic acid, no clear deliquescence point could be identified and obvious water uptake started at 75% RH. ZSR predicted values were in fair agreement with the measured values at low RH, but slightly lower above the DRH of KCl.

Brooks *et al*.^25^ studied hygroscopic behaviors of four humic acids (Fluka HA, Pahokee Peat Reference HA, Leonardite Standard HA, Suwannee River Reference FA) and corresponding internal mixtures with ammonium sulfate using the HTDMA. They also found that water uptake of mixed humic acid/ammonium sulfate particles was consistent with predictions assuming individual component took up water independently, i.e. the ZSR rule. Badger *et al*.^50^ measured hygroscopic properties of mixed particles containing ammonium sulfate with humic acid (Aldrich HA or Leonardite Standard HA) by combining HTDMA with Fourier transform infra red spectroscopy. Their results indicated that humic acid could lower the DRH of ammonium sulfate and cause slight water uptake of mixed particles prior to deliquescence of ammonium sulfate. In addition, the measured growth factors of mixed particles also agreed well with the ZSR predictions.

### Potassium Chloride/Oxalic Acid/Levoglucosan Mixtures

**Figure 5:**
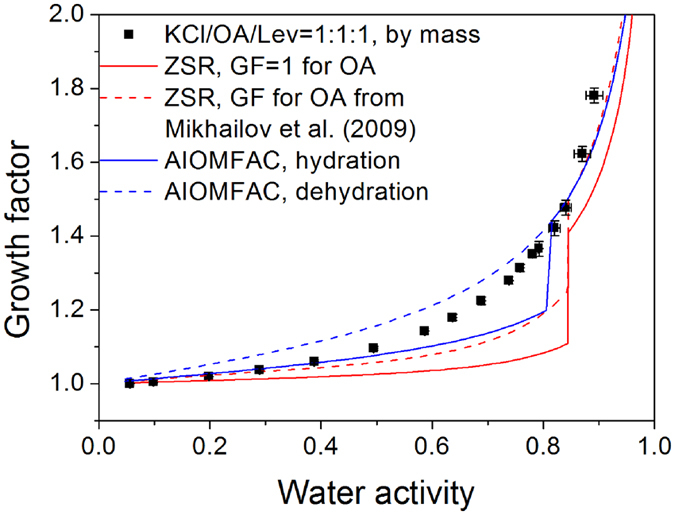
Hygroscopic growth factors of 1:1:1 KCl/OA/Lev mixed aerosols as a function of water activity. Hygroscopic growth curves from the ZSR and AIOMFAC model are indicated by red and blue lines, respectively. The ZSR predictions based on GFs of OA from Mikhailov *et al*.^37^ are also shown, indicated by red dashed line. For AIOMFAC evaluations, the hydration curve is derived assuming KCl remained solid in the initial particles and organics were in a liquid state while the dehydration one assuming all the mixed species remained liquid in the RH range studied.

Considering the difference of hygroscopic properties between organics, we determined the influences of multicomponent organics composed of oxalic acid and levoglucosan (OA/Lev) on the hygroscopicity of KCl. As shown in Fig. 5, the mixed particles containing KCl, oxalic acid and levoglucosan with equal mass ratio took up water gradually in the whole RH range, suggesting no apparent phase change occurred. It was obvious that hydration curves predicted from AIOMFAC and ZSR method deviated a lot from the hygroscopic growth in the RH range of 50–80%. This is likely caused by the fact that water content associated with organic components enhanced partial dissolution of KCl at medium RH. The predicted DRH from AIOMFAC was 81% RH compared to the continuous water uptake behavior of mixed particles. After full deliquescence of KCl, the predictions from AIOMFAC and ZSR methods based on GFs for oxalic acid from Mikhailov *et al*.^37^ were close to hygroscopic growth of mixed particles. To better describe the hygroscopic growth of equal mass KCl/OA/Lev mixed particles, the dehydration curve from AIOMFAC was also given for comparison. It can be seen that water contents predicted by the model was higher than the measurements at low and medium RH, suggesting KCl may not be completely dissolved in this RH range. In our previous study, the ternary mixture containing ammonium sulphate, oxalic acid and levoglucosan also showed similar hygroscopic behavior, which was beyond the prediction ability of thermodynamic model^39^. The possible reason is that organic mixture has more complex consequences for the hygroscopic behavior of KCl than single organic species. The KCl aerosols internally mixed with oxalic acid or levoglucosan at equal mass ratio exhibit prompt deliquescence transitions, albeit at a lower RH relative to pure potassium chloride. The continuous water uptake of equal mass KCl/OA/Lev mixture from low RH highlights the role of levoglucosan in the multicomponent mixed system. Levoglucosan is a minor component in the equal mass KCl/OA/Lev mixture compared to the major components including KCl and oxalic acid with high deliquescence points. Our previous study has shown that levoglucosan could suppress crystallization of oxalic acid in the initial particles resulting in more water uptake at low RH^39^. The water uptake contributed by levoglucosan and oxalic acid at low and medium RH further promotes partial dissolution of KCl. Consequently, levoglucosan could dramatically affect the hygroscopic behavior of KCl in the medium RH range by altering phase state of oxalic acid.

### Effect of Organic Species on the Hygroscopic Properties of Inorganic Salt

The GFs including both measurements and ZSR predictions of mixed particles at 90% RH were used to calculate the *ξ*′_*w*_ value. As can be seen from Table 2, interactions between oxalic acid and potassium chloride have a great effect on the equal mass mixture due to the dissolution of oxalic acid in aqueous phase, as indicated by a *ξ*′_*w*_ value of 1.86. Similarly, the *ξ*′_*w*_ value of 1.62 for equal mass KCl/OA/Lev mixed particles also implies the water uptake contributed by oxalic acid at high RH. As for the mixtures containing KCl with humic acid or levoglucosan, the *ξ*′_*w*_ values do not deviate much from 1 regardless of different composition ratio, indicating there are no obvious enhancing or reducing effects on hygroscopic growth of mixed particles. Although the 3:1 KCl/levoglucosan mixed particles have a ξ′_w_ value of 1.32 slightly larger than 1, this value still does not deviate much from 1. As shown in Fig. 3a, the general agreement between measured hygroscopic growth and ZSR predicted curve for 3:1 KCl/Lev mixed particles at high RH above 80% suggests no obvious effects of interaction between mixed species on the water uptake. Chan and Chan^31^ found that interactions between fulvic acid and ammonium sulfate could enhance the water uptake at 90% RH while this enhancement effect was not observed for fulvic acid/sodium chloride mixture at the same RH.

Although the potassium chloride has a high deliquescence point at 84% RH, the water soluble organic compounds such as oxalic acid, levoglucosan and humic acid in the mixture can reduce the deliquescence point of KCl and promote water uptake even at low RH. The water uptake behaviors of KCl-organic mixed particles are similar to those observed for ambient biomass smoke aerosols. Boreddy *et al*. observed that hygroscopic growth of water-soluble matter extracted from biomass burning aerosols in east Africa was well correlated with mass fractions of K^+^, Cl^+^, and organic carbon such as levoglucosan and diacids within particles^51^. They attributed lower growth factors obtained over the sampling site to the formation of less water-soluble potassium oxalate (K_2_C_2_O_4_) during atmospheric aging, which was supported by our observations for KCl aerosols with dominant oxalic acid content. Semeniuk *et al*. reported the mixed organic–inorganic particles from young smoke of flaming and smoldering fires in southern Africa took up water dramatically between 55 and 100% RH, depending on the chemical composition of water-soluble species^52^. More water content in aerosol particles under low and medium humidity conditions may contribute to the aging of potassium chloride, leading to formation of nitrate and sulfate through the heterogeneous reaction with SO_2_, NO_x_ 15. The particulates from biomass burning emissions directly contribute to haze pollution. Additionally, particulate matter can take up water under ambient RH conditions providing aqueous medium for heterogeneous reactions, which further aggravate the haze pollution.

## Methods

### Sample Preparation

Physicochemical properties and mixing ratio of the compounds investigated were given in Tables 1 and 3. The aerosol particles were generated from a constant output atomizer (1500, MSP) containing aqueous solutions with a mass concentration of 0.1%. The dilute solutions were prepared by dissolving each pure component or mixture in ultrapure water (EASY Pure^®^ II UF ultrapure water system, 18.2 MΩ cm). Due to its complex composition, humic acid could not be completely dissolved in the ultrapure water. The humic acid suspensions were filtered through the filter paper (Whatman, medium speed) to remove insoluble material. In order to accurately calibrate the concentration of humic acid, subsequent processing were performed with the clarified filtrate. The organic elemental analysis indicated that mass fraction of carbon, hydrogen, nitrogen in solid humic acid (Aldrich) samples were 41.32%, 3.29%, 1.21%, respectively. Carbon content (g ml^−1^) in the humic acid solution was measured using a total organic carbon analyzer (TOC, Analytikjena multi N/C 2100). The concentration of HA is obtained as follows: Concentration of HA (g ml^−1^) = carbon element concentration of the solution measured by TOC (g ml^−1^)/mass fraction of carbon element in solid HA samples. The humic acid solution with specific concentration was used to prepare mixture solution containing KCl.

### Hygroscopic Growth Measurements

The hygroscopicity tandem differential mobility analyzer (HTDMA) has been used in our early studies^39^,^53^,^54^. Only a brief description is given here. The generated aerosols were dried to RH < 5% by passing through silica gel diffusion dryers combined with a Nafion gas dryer (Perma Pure Inc., USA). The aerosol particles at a flow rate of 0.3 L min^−1^ were charged first and then entered the first differential mobility analyzer (DMA1). After size selection by DMA1, the polydisperse aerosol particles were transformed into nearly monodisperse ones with the particle diameter of 100 nm. The size-selected particles then passed through the humidity conditioner containing two Nafion humidification tubes, where the aerosol flow was humidified to a desired RH with a residence time of approximate 5 s. The size distribution of monodisperse aerosol particles after exposing to varying RHs was determined by the second differential mobility analyzer (DMA2) and a condensation particle counter (CPC, MSP 1500). The inversion of HTDMA measurement data was based on a log-normal size distribution approximation^55^. The humidity of sheath flow at the outlet of DMA2 was monitored using a dew point hygrometer (Michell, UK) accurate to within ±0.08% RH and ±0.1 K. The experiments were conducted at room temperature (297 ± 1 K).

The ratio of the diameter of particles after humidification at given RH relative to that at RH < 5% is defined as hygroscopic growth factor (GF) in this study. The humidity sensors used in the HTDMA were calibrated by measuring deliquescence points of three salts including sodium chloride (NaCl), ammonium sulfate (NH_4_)_2_SO_4_ and potassium chloride (KCl). The comparisons of measured DRHs and theory predictions showed the measurement uncertainty for DRHs of these three salts was within ±1.5% RH. The uncertainty in measured RH was thus estimated to be within ±1.5% RH. All measured growth factors at a given RH are the average values of at least three repeated measurements. The corresponding uncertainty in GFs is represented by standard deviation, which is typically within 0.02. The performance of the HTDMA system was verified by measuring the hygroscopic growth of ammonium sulfate during humidification. The measured deliquescence point (80 ± 0.5% RH) and GFs (GF = 1.44 and 1.68 at 80% and 90% RH for 100 nm particles, respectively) for ammonium sulfate were consistent with the reported values (DRH = 80 ± 1.2% RH, GF = 1.46 and 1.68 at 80% and 90% RH for 100 nm particles, respectively) in the literature^56^,^57^.

### Model Methods

Due to the significant curvature effect, the relation between water activity *a*_*w*_ and RH for submicron droplets should be expressed by Köhler equation:


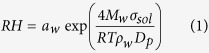


Here, *M*_*w*_ is the molar mass of water, *σ*_*sol*_ is the surface tension of the droplet, *R* is universal gas constant, *T* is temperature, *ρ*_*w*_ is the density of water, *D*_*p*_ is the droplet diameter. As an approximation, the surface tension of pure water (0.072 J m^−2^) was used in equation (1). Although organics may reduce the surface tension of the droplet, *a*_*w*_ error caused by approximation was negligible compared with the uncertainty of the RH measurement^40^.

According to the method proposed by Kreidenweis *et al*.^58^, the continuous hygroscopic growth of single species can be well described by a three-parameter fit equation:


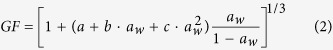


The coefficients *a, b* and *c* obtained by Eq. (2) fitting to GF-*a*_w_ measurement data are given in Table 4. For oxalic acid, the corresponding coefficients from Mikhailov *et al*.^37^ are also included in Table 4.

The ZSR method is a valuable tool to predict hygroscopic growth of multicomponent aerosol particles. Assuming each component in the mixture takes up water independently, hygroscopic growth factor (*GF*_*mixed*_) of internally mixed particles can be expressed as follows^24^,^59^:


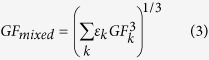


where *GF*_*k*_ is hygroscopic growth factor of pure component *k, ε*_*k*_ is the corresponding volume fraction in the dry mixture, given by:


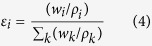


where *w*_*i*_ is the mass fraction of component *i, ρ*_*i*_ is the corresponding density. The ZSR relation is based on the assumption that no interactions exist between species in the mixture thus the water uptake by mixed particles is equal to that by the sum of the individual components. It has been confirmed that this simple rule could accurately estimate hygroscopic growth of atmospheric inorganic/organic mixtures at high RH except for a few cases^24^,^28^,^57^. In this study, the hydration curve of KCl predicted by the AIOMFAC model and the three-parameter fit curve of WSOCs were applied in the ZSR calculations.

Predictions from Aerosol Inorganic-Organic Mixtures Functional groups the Activity Coefficients (AIOMFAC) model are also used to compare with our data from HTDMA measurements. The AIOMFAC model is developed for calculations of the activity coefficients in organic and/or inorganic mixtures with different concentration conditions from simple binary to complex multicomponent systems which are the major constituents of atmospheric particles^36^,^42^. AIOMFAC based on group-contribution method can describe non-ideal mixing behavior of organic-inorganic mixture in solutions and treat interactions between mixture components which may result in significant deviations from ideal mixing.

To quantitatively evaluate the effect of organic matter on the hygroscopic growth of inorganic salt, Cruz and Pandis^22^ proposed a parameter *ξ*_*w*_ to describe this effect:


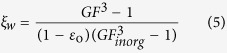


Here *ξ*_*w*_ is the ratio of water volume uptake by mixed aerosols to that by inorganic component, *GF, GF*_*inorg*_ is the hygroscopic growth factor of the mixture and inorganic component, respectively, *ε*_*o*_ is the volume fraction of organic matter in the mixture. This expression assumes water uptake of mixed particle is only contributed by the inorganic component. In fact, some water soluble organic compounds display obvious hygroscopic growth at high RH. In order to accurately assess the effects of interaction between mixed species on the hygroscopic properties, water uptake by each component should be taken into consideration and thus equation (5) can be modified as:


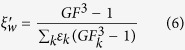


where *ξ*′_*w*_ is the ratio of water volume uptake by mixed aerosols to the sum of water volume uptake by individual component, *GF*_*k*_ is the hygroscopic growth factor of pure component *k, ε*_*k*_ is the volume fraction of the pure component *k* in the dry particle.

## Additional Information

**How to cite this article**: Jing, B. *et al*. Hygroscopic properties of potassium chloride and its internal mixtures with organic compounds relevant to biomass burning aerosol particles. *Sci. Rep.* **7**, 43572; doi: 10.1038/srep43572 (2017).

**Publisher's note:** Springer Nature remains neutral with regard to jurisdictional claims in published maps and institutional affiliations.

## Figures and Tables

**Table 1:** Comparisons of DRH for mixtures containing potassium chloride (KCl) with oxalic acid (OA) or levoglucosan (Lev) between measurements and predictions from AIOMFAC.

Mixture by mass ratio	DRH/%	
	Measurement	AIOMFAC prediction
3:1 KCl/OA	79 ± 1	82.6
1:1 KCl/OA	79 ± 1	79.0
1:3 KCl/OA	n.o.^a^	68.2
3:1 KCl/Lev	80 ± 1	84.0
1:1 KCl/Lev	79 ± 1	84.3
1:3 KCl/Lev	n.o.^a^	85.2

^a^Not observed (n.o.).

**Table 2:** Growth factor (GF) and *ξ*_*w*_’ for potassium chloride (KCl) aerosols internally mixed with organic compounds at 90% RH.

Mixture by mass ratio	GF at 90% RH		*ξ*_*w*_′ at 90% RH
	Measurement	ZSR prediction	
3:1 KCl/OA	1.93	1.82	1.23
1:1 KCl/OA	1.85	1.57	1.86
1:3 KCl/OA	1.35	1.30	1.22
3:1 KCl/Lev	2.08	1.92	1.32
1:1 KCl/Lev	1.82	1.76	1.13
1:3 KCl/Lev	1.58	1.59	0.97
3:1 KCl/HA	1.89	1.92	0.95
1:1 KCl/HA	1.79	1.77	1.04
1:3 KCl/HA	1.69	1.62	1.18
1:1:1 KCl/OA/Lev	1.78	1.57	1.62

For oxalic acid, the GF = 1 was used in the ZSR rule.

**Table 3:** Chemical properties of species studied in this work.

Substance	Chemical Formula	MW (g mol^−1^)	ρ (g cm^−3^)	Supplier/Purity
potassium chloride	KCl	74.55	1.988	Alfa Aesar, 99.997%
Levoglucosan	C_6_H_10_O_5_	162.1	1.62	Aldrich, 99%
Oxalic acid	C_2_H_2_O_4_	90	1.9	Aldrich, 99.999%
Oxalic acid (dihydrate)	C_2_H_2_O_4_·2H_2_O	126	1.65	—
Humic acid	**—**	**—**	1.5	Aldrich

**Table 4:** The fitting parameters of the hygroscopic growth curve for the pure organic aerosol particles with the Eq. (2).

Substance	*a*	*b*	*c*	R^2^
Oxalic acid	0.6185^a^	−1.2315^a^	0.9511^a^	0.9952^a^
Levoglucosan	0.4315	−0.4929	0.2789	0.9997
Humic acid	0.2525	0.0806	−0.0797	0.9941

^a^From Mikhailov *et al*.^37^.

## References

[b1] Haywood J. & Boucher O. Estimates of the direct and indirect radiative forcing due to tropospheric aerosols: A review. Rev. Geophys. 38, 513–543, doi: 10.1029/1999rg000078 (2000).

[b2] Saxena P. & Hildemann L. M. Water-soluble organics in atmospheric particles: A critical review of the literature and application of thermodynamics to identify candidate compounds. J. Atmos. Chem. 24, 57–109, doi: 10.1007/bf00053823 (1996).

[b3] Zhang Q. . Ubiquity and dominance of oxygenated species in organic aerosols in anthropogenically-influenced Northern Hemisphere midlatitudes. Geophys. Res. Lett. 34, n/a–n/a, doi: 10.1029/2007gl029979 (2007).

[b4] Decesari S. . The water-soluble organic component of size-segregated aerosol, cloud water and wet depositions from Jeju Island during ACE-Asia. Atmos. Environ. 39, 211–222, doi: 10.1016/j.atmosenv.2004.09.049 (2005).

[b5] Ansari A. S. & Pandis S. N. Water absorption by secondary organic aerosol and its effect on inorganic aerosol behavior. Environ. Sci. Technol. 34, 71–77, doi: 10.1021/es990717q (2000).

[b6] Andreae M. O. & Merlet P. Emission of trace gases and aerosols from biomass burning. Global Biogeochem. Cycles 15, 955–966, doi: 10.1029/2000gb001382 (2001).

[b7] Cheng Y. . Biomass burning contribution to Beijing aerosol. Atmos. Chem. Phys. 13, 7765–7781, doi: 10.5194/acp-13-7765-2013 (2013).

[b8] Reid J. S., Koppmann R., Eck T. F. & Eleuterio D. P. A review of biomass burning emissions part II: intensive physical properties of biomass burning particles. Atmos. Chem. Phys. 5, 799–825 (2005).

[b9] Crutzen P. J. & Andreae M. O. Biomass burning in the tropics - impact on atmospheric chemistry and biogeochemical cycles. Science 250, 1669–1678, doi: 10.1126/science.250.4988.1669 (1990). 17734705

[b10] Dusek U. . Water uptake by biomass burning aerosol at sub- and supersaturated conditions: closure studies and implications for the role of organics. Atmos. Chem. Phys. 11, 9519–9532, doi: 10.5194/acp-11-9519-2011 (2011).

[b11] Hand J. L. . Measured and modeled humidification factors of fresh smoke particles from biomass burning: role of inorganic constituents. Atmos. Chem. Phys. 10, 6179–6194, doi: 10.5194/acp-10-6179-2010 (2010).

[b12] Posfai M., Simonics R., Li J., Hobbs P. V. & Buseck P. R. Individual aerosol particles from biomass burning in southern Africa: 1. Compositions and size distributions of carbonaceous particles. J. Geophys. Res.- Atmos. 108, doi: 10.1029/2002jd002291 (2003).

[b13] Rissler J. . Size distribution and hygroscopic properties of aerosol particles from dry-season biomass burning in Amazonia. Atmos. Chem. Phys. 6, 471–491 (2006).

[b14] Li J. Individual aerosol particles from biomass burning in southern Africa: 2, Compositions and aging of inorganic particles. J. Geophys. Res. 108, doi: 10.1029/2002jd002310 (2003).

[b15] Zauscher M. D., Wang Y., Moore M. J., Gaston C. J. & Prather K. A. Air quality impact and physicochemical aging of biomass burning aerosols during the 2007 San Diego wildfires. Environ. Sci. Technol. 47, 7633–7643, doi: 10.1021/es4004137 (2013). 23750590

[b16] Freney E. J., Martin S. T. & Buseck P. R. Deliquescence and efflorescence of potassium salts relevant to biomass-burning aerosol particles. Aerosol Sci. Technol. 43, 799–807, doi: 10.1080/02786820902946620 (2009).

[b17] Rissler J. . Hygroscopic behavior of aerosol particles emitted from biomass fired grate boilers. Aerosol Sci. Technol. 39, 919–930, doi: 10.1080/02786820500331068 (2005).

[b18] Fuzzi S. . A simplified model of the water soluble organic component of atmospheric aerosols. Geophys. Res. Lett. 28, 4079–4082, doi: 10.1029/2001gl013418 (2001).

[b19] Kawamura K., Barrie L. A. & Toom-Sauntry D. Intercomparison of the measurements of oxalic acid in aerosols by gas chromatography and ion chromatography. Atmos. Environ. 44, 5316–5319, doi: 10.1016/j.atmosenv.2010.08.051 (2010).

[b20] Simoneit B. R. T. . Levoglucosan, a tracer for cellulose in biomass burning and atmospheric particles. Atmos. Environ. 33, 173–182, doi: 10.1016/s1352-2310(98)00145-9 (1999).

[b21] Decesari S. . Chemical features and seasonal variation of fine aerosol water-soluble organic compounds in the Po Valley, Italy. Atmos. Environ. 35, 3691–3699, doi: 10.1016/s1352-2310(00)00509-4 (2001).

[b22] Cruz C. N. & Pandis S. N. Deliquescence and hygroscopic growth of mixed inorganic-organic atmospheric aerosol. Environ. Sci. Technol. 34, 4313–4319, doi: 10.1021/es9907109 (2000).

[b23] Peng C., Chan M. N. & Chan C. K. The hygroscopic properties of dicarboxylic and multifunctional acids: Measurements and UNIFAC predictions. Environ. Sci. Technol. 35, 4495–4501, doi: 10.1021/es0107531 (2001). 11757607

[b24] Prenni A. J., De Mott P. J. & Kreidenweis S. M. Water uptake of internally mixed particles containing ammonium sulfate and dicarboxylic acids. Atmos. Environ. 37, 4243–4251, doi: 10.1016/s1352-2310(03)00559-4 (2003).

[b25] Brooks S. D., DeMott P. J. & Kreidenweis S. M. Water uptake by particles containing humic materials and mixtures of humic materials with ammonium sulfate. Atmos. Environ. 38, 1859–1868, doi: 10.1016/j.atmosenv.2004.01.009 (2004).

[b26] Gysel M. . Hygroscopic properties of water-soluble matter and humic-like organics in atmospheric fine aerosol. Atmos. Chem. Phys. 4, 35–50, doi: 10.5194/acp-4-35-2004 (2004).

[b27] Svenningsson B. . Hygroscopic growth and critical supersaturations for mixed aerosol particles of inorganic and organic compounds of atmospheric relevance. Atmos. Chem. Phys. 6, 1937–1952 (2006).

[b28] Zardini A. A. . A combined particle trap/HTDMA hygroscopicity study of mixed inorganic/organic aerosol particles. Atmos. Chem. Phys. 8, 5589–5601 (2008).

[b29] Zamora I. R. & Jacobson M. Z. Measuring and modeling the hygroscopic growth of two humic substances in mixed aerosol particles of atmospheric relevance. Atmos. Chem. Phys. 13, 8973–8989, doi: 10.5194/acp-13-8973-2013 (2013).

[b30] Choi M. Y. & Chan C. K. The effects of organic species on the hygroscopic behaviors of inorganic aerosols. Environ. Sci. Technol. 36, 2422–2428, doi: 10.1021/es0113293 (2002). 12075799

[b31] Chan M. N. & Chan C. K. Hygroscopic properties of two model humic-like substances and their mixtures with inorganics of atmospheric importance. Environ. Sci. Technol. 37, 5109–5115, doi: 10.1021/es034272o (2003). 14655696

[b32] Ma Q., Ma J., Liu C., Lai C. & He H. Laboratory study on the hygroscopic behavior of external and internal C-2-C-4 dicarboxylic acid-NaCl mixtures. Environ. Sci. Technol. 47, 10381–10388, doi: 10.1021/es4023267 (2013). 23941508

[b33] Drozd G., Woo J., Hakkinen S. A. K., Nenes A. & McNeill V. F. Inorganic salts interact with oxalic acid in submicron particles to form material with low hygroscopicity and volatility. Atmos. Chem. Phys. 14, 5205–5215, doi: 10.5194/acp-14-5205-2014 (2014).

[b34] Carrico C. M. . Water uptake and chemical composition of fresh aerosols generated in open burning of biomass. Atmos. Chem. Phys. 10, 5165–5178, doi: 10.5194/acp-10-5165-2010 (2010).

[b35] Tang I. N. Thermodynamic and optical properties of mixed-salt aerosols of atmospheric importance. J. Geophys. Res.- Atmos. 102, 1883–1893, doi: 10.1029/96jd03085 (1997).

[b36] Zuend A., Marcolli C., Luo B. P. & Peter T. A thermodynamic model of mixed organic-inorganic aerosols to predict activity coefficients. Atmos. Chem. Phys. 8, 4559–4593 (2008).

[b37] Mikhailov E., Vlasenko S., Martin S. T., Koop T. & Poeschl U. Amorphous and crystalline aerosol particles interacting with water vapor: conceptual framework and experimental evidence for restructuring, phase transitions and kinetic limitations. Atmos. Chem. Phys. 9, 9491–9522 (2009).

[b38] Prenni A. J. . The effects of low molecular weight dicarboxylic acids on cloud formation. J. Phys. Chem. A 105, 11240–11248, doi: 10.1021/jp012427d (2001).

[b39] Jing B. . Hygroscopic behavior of multicomponent organic aerosols and their internal mixtures with ammonium sulfate. Atmos. Chem. Phys. 16, 4101–4118, doi: 10.5194/acp-16-4101-2016 (2016).

[b40] Mochida M. & Kawamura K. Hygroscopic properties of levoglucosan and related organic compounds characteristic to biomass burning aerosol particles. J. Geophys. Res.- Atmos. 109, doi: 10.1029/2004jd004962 (2004).

[b41] Koehler K. A. . Water activity and activation diameters from hygroscopicity data - Part II: Application to organic species. Atmos. Chem. Phys. 6, 795–809 (2006).

[b42] Zuend A. . New and extended parameterization of the thermodynamic model AIOMFAC: calculation of activity coefficients for organic-inorganic mixtures containing carboxyl, hydroxyl, carbonyl, ether, ester, alkenyl, alkyl, and aromatic functional groups. Atmos. Chem. Phys. 11, 9155–9206, doi: 10.5194/acp-11-9155-2011 (2011).

[b43] Zamora I. R., Tabazadeh A., Golden D. M. & Jacobson M. Z. Hygroscopic growth of common organic aerosol solutes, including humic substances, as derived from water activity measurements. J. Geophys. Res.- Atmos. 116, doi: 10.1029/2011jd016067 (2011).

[b44] Wu Z. J., Nowak A., Poulain L., Herrmann H. & Wiedensohler A. Hygroscopic behavior of atmospherically relevant water-soluble carboxylic salts and their influence on the water uptake of ammonium sulfate. Atmos. Chem. Phys. 11, 12617–12626, doi: 10.5194/acp-11-12617-2011 (2011).

[b45] Brooks S. D., Wise M. E., Cushing M. & Tolbert M. A. Deliquescence behavior of organic/ammonium sulfate aerosol. Geophys. Res. Lett. 29, doi: 10.1029/2002gl014733 (2002).

[b46] Peng C. G. & Chan C. K. The water cycles of water-soluble organic salts of atmospheric importance. Atmos. Environ. 35, 1183–1192, doi: 10.1016/s1352-2310(00)00426-x (2001).

[b47] Ghorai S., Wang B., Tivanski A. & Laskin A. Hygroscopic properties of internally mixed particles composed of NaCl and water-soluble organic acids. Environ. Sci. Technol. 48, 2234–2241, doi: 10.1021/es404727u (2014). 24437520

[b48] Parsons M. T., Knopf D. A. & Bertram A. K. Deliquescence and crystallization of ammonium sulfate particles internally mixed with water-soluble organic compounds. J. Phys. Chem. A 108, 11600–11608, doi: 10.1021/jp0462862 (2004).

[b49] Ling T. Y. & Chan C. K. Partial crystallization and deliquescence of particles containing ammonium sulfate and dicarboxylic acids. J. Geophys. Res.- Atmos. 113, doi: 10.1029/2008jd009779 (2008).

[b50] Badger C. L. . Phase transitions and hygroscopic growth of aerosol particles containing humic acid and mixtures of humic acid and ammonium sulphate. Atmos. Chem. Phys. 6, 755–768 (2006).

[b51] Boreddy S. K. R., Kawamura K., Mkoma S. & Fu P. Hygroscopic behavior of water-soluble matter extracted from biomass burning aerosols collected at a rural site in Tanzania, East Africa. J. Geophys. Res.- Atmos. 119, 12233–12245, doi: 10.1002/2014jd021546 (2014).

[b52] Semeniuk T. A., Wise M. E., Martin S. T., Russell L. M. & Buseck P. R. Hygroscopic behavior of aerosol particles from biomass fires using environmental transmission electron microscopy. J. Atmos. Chem. 56, 259–273, doi: 10.1007/s10874-006-9055-5 (2007).

[b53] Peng C., Jing B., Guo Y. C., Zhang Y. H. & Ge M. F. Hygroscopic behavior of multicomponent aerosols involving NaCl and dicarboxylic acids. J. Phys. Chem. A 120, 1029–1038, doi: 10.1021/acs.jpca.5b09373 (2016). 26820230

[b54] Liu Q. . Hygroscopicity of internally mixed multi-component aerosol particles of atmospheric relevance. Atmos. Environ. 125, 69–77, doi: 10.1016/j.atmosenv.2015.11.003 (2016).

[b55] Stolzenburg M. R. & McMurry P. H. Equations governing single and tandem DMA configurations and a new lognormal approximation to the transfer function. Aerosol Sci. Technol. 42, 421–432, doi: 10.1080/02786820802157823 (2008).

[b56] Gysel M., Weingartner E. & Baltensperger U. Hygroscopicity of aerosol particles at low temperatures. 2. Theoretical and experimental hygroscopic properties of laboratory generated aerosols. Environ. Sci. Technol. 36, 63–68, doi: 10.1021/es010055g (2002). 11811491

[b57] Sjogren S. . Hygroscopic growth and water uptake kinetics of two-phase aerosol particles consisting of ammonium sulfate, adipic and humic acid mixtures. J. Aerosol Sci. 38, 157–171, doi: 10.1016/j.jaerosci.2006.11.005 (2007).

[b58] Kreidenweis S. M. . Water activity and activation diameters from hygroscopicity data - Part I: Theory and application to inorganic salts. Atmos. Chem. Phys. 5, 1357–1370 (2005).

[b59] Stokes R. H. & Robinson R. A. Interactions in aqueous nonelectrolyte solutions. I. Solute-solvent equilibria. J. Phys. Chem. 70, 2126–2131, doi: 10.1021/j100879a010 (1966).

